# Supplementary material for: Analytical method for detecting outlier evaluators
Source: BMC Med Res Methodol. 2023 Aug 1;23:177. doi: 10.1186/s12874-023-01988-4 (PMC10391872; doi:10.1186/s12874-023-01988-4)
Supplement: Supplementary file 1 — Additional file 1. [file 12874_2023_1988_MOESM1_ESM.pdf]

1 Hypothesis testing for truncated mean

A more robust approach is to compute a truncated mean of the coefficients where potential 'outliers' can be prevented from contaminating the average effect. Let $\beta_{(1)}, \beta_{(2)}, \ldots, \beta_{(M)}$ be the ordered values of the regression coefficients. A $\delta \times 100\%$ truncated mean can be calculated as follows [?]:

$$\overline{\beta}_{\text{truncated}} = \frac{1}{M - 2[M \cdot \delta]} \sum_{q=[M\cdot\delta]+1}^{M-[M\cdot\delta]} \beta_{(q)}, \tag{1}$$

where $[x]$ denotes the integer part of x.

The null hypothesis that the j-th evaluator is not an 'outlier' is now to compare the regression coefficient of the j-th evaluator to the $\delta \times 100\%$ truncated mean:

$$H_{0,j} : \beta_j - \overline{\beta}_{\text{truncated}} = 0, \quad j = 1, 2, \ldots, M. \tag{2}$$

Let the set of the regression coefficients that are truncated be $\mathcal{A} = \{\beta_{(1)}, \ldots, \beta_{([M\cdot\delta])}, \beta_{(M-[M\cdot\delta]+1)}, \ldots, \beta_{(M)}\}$. It follows that the l-th, $l = 1, \ldots, M$, element of the contrast matrix for testing $H_{0,j}$ is

$$L_{\delta\times 100\%, jl} = \begin{cases} 0, & \text{If } \beta_l \in \mathcal{A} \text{ and } l \neq j \\ 1, & \text{If } \beta_l \in \mathcal{A} \text{ and } l = j \\ -\frac{1}{M-2[M\cdot\delta]}, & \text{If } \beta_l \notin \mathcal{A} \text{ and } l \neq j \\ 1 - \frac{1}{M-2[M\cdot\delta]}, & \text{If } \beta_l \notin \mathcal{A} \text{ and } l = j, \end{cases} \tag{3}$$

and the null hypothesis $H_{0,j}$ can be written as $H_{0,j} : \boldsymbol{L}_{\delta\times 100\%,j}^T \boldsymbol{\beta} = 0$. Note that, we use $\boldsymbol{L}_{\delta\times 100\%,j}, j = 1, \ldots, M$, to denote the contrast matrix, indicating that the hypothesis test is comparing each evaluator's regression coefficient with the $\delta \times 100\%$ truncated mean of the regression coefficients of all evaluators.

The Wald test statistic under the null hypothesis is:

$$\left(\boldsymbol{L}_{\delta\times 100\%,j}^T \widehat{\boldsymbol{\beta}}\right)^T \left[\boldsymbol{L}_{\delta\times 100\%,j}^T \widehat{\Sigma} \boldsymbol{L}_{\delta\times 100\%,j}\right]^{-1} \left(\boldsymbol{L}_{\delta\times 100\%,j}^T \widehat{\boldsymbol{\beta}}\right) \xrightarrow{D} \chi_1^2. \tag{4}$$

Note that the contrast matrix $\boldsymbol{L}_{\delta\times 100\%,j}$ is not directly available since we need to know the ordering of the true coefficients $\beta_1, \ldots, \beta_M$ in advance. An approximation to $\boldsymbol{L}_{\delta\times 100\%,j}$ can be based on the estimated regression coefficients $\widehat{\beta}_1, \ldots, \widehat{\beta}_M$; that is,

$$L_{\delta\times 100\%, jl} \approx \begin{cases} 0, & \text{If } \widehat{\beta}_l \in \mathcal{A}^* \text{ and } l \neq j \\ 1, & \text{If } \widehat{\beta}_l \in \mathcal{A}^* \text{ and } l = j \\ -\frac{1}{M-2[M\cdot\delta]}, & \text{If } \widehat{\beta}_l \notin \mathcal{A}^* \text{ and } l \neq j \\ 1 - \frac{1}{M-2[M\cdot\delta]}, & \text{If } \widehat{\beta}_l \notin \mathcal{A}^* \text{ and } l = j, \end{cases} \tag{5}$$

where $\mathcal{A}^* = \{\widehat{\beta}_{(1)}, \ldots, \widehat{\beta}_{([M\cdot\delta])}, \widehat{\beta}_{(M-[M\cdot\delta]+1)}, \ldots, \widehat{\beta}_{(M)}\}$.

2 False discovery rate estimation

Let $\boldsymbol{R}$ denote the total number of null hypotheses being rejected (i.e. discoveries) among $H_{0,1}, \ldots, H_{0,M}$ and $\boldsymbol{V}$ denote the number of rejected true null hypotheses (i.e. false discoveries). Define the ratio as

$$\boldsymbol{Q} = \begin{cases} \frac{\boldsymbol{V}}{\boldsymbol{R}}, & \text{If } \boldsymbol{R} > 0, \\ 0, & \text{If } \boldsymbol{R} = 0. \end{cases} \tag{6}$$

Then, the FDR is the expectation of false discoveries among discoveries: $\mathrm{E}(\boldsymbol{Q})$. Therefore, in the context of our paper, given a power ϕ, the FDR is:

$$\mathrm{E}(\boldsymbol{Q}; \phi) = \mathrm{E}\left(\frac{\boldsymbol{V}(\phi)}{\boldsymbol{R}(\phi)}\right). \tag{7}$$

Note that we use the notations $\mathrm{E}(\boldsymbol{Q}; \phi)$, $\boldsymbol{V}(\phi)$, $\boldsymbol{R}(\phi)$ to indicate that they are dependent on the pre-specified power ϕ of the test.

Storey and Tibshirani provided an approximation formula for the FDR:

$$\mathrm{E}(\boldsymbol{Q}; \phi) = \mathrm{E}\left(\frac{\boldsymbol{V}(\phi)}{\boldsymbol{R}(\phi)}\right) \approx \frac{\mathrm{E}(\boldsymbol{V}(\phi))}{\mathrm{E}(\boldsymbol{R}(\phi))}. \tag{8}$$

Recall that given a particular power ϕ, the corresponding significance levels for hypotheses $H_{0,1}, H_{0,2}, \ldots, H_{0,M}$ are $\alpha_1(\phi), \alpha_2(\phi), \ldots, \alpha_M(\phi)$, respectively. Since $\alpha_j(\phi)$ represents the probability of falsely rejecting $H_{0,j}$ given it is true, the numerator $\mathrm{E}(\boldsymbol{V}(\phi))$ in (8) can be written as

$$\mathrm{E}(\boldsymbol{V}(\phi)) = \sum_{j \in \mathcal{T}} \alpha_j(\phi), \tag{9}$$

where $\mathcal{T}$ is the set of the indexes of the true null hypotheses (i.e. 'normal' evaluators). However, the set of true null hypotheses are unknown. We make the assumption of rare 'outlier' evaluators; that is $\sum_{j \in \mathcal{T}} \alpha_j(\phi) \approx \sum_{j=1}^{M} \alpha_j(\phi)$. Therefore, the numerator of (8) can be approximated by

$$\mathrm{E}(\boldsymbol{V}(\phi)) \approx \sum_{j=1}^{M} \alpha_j(\phi). \tag{10}$$

A simple estimate of the denominator $\mathrm{E}(\boldsymbol{R}(\phi))$ is the observed total number of rejected null hypotheses from an experiment; that is,

$$\mathrm{E}(\boldsymbol{R}(\phi)) \approx \boldsymbol{R}(\phi) = \sum_{j=1}^{M} \mathrm{I}(p_j < \alpha_j(\phi)), \tag{11}$$

where p_j is the p-value corresponding to the j-th evaluator.

Plugging (10) and (11) into Formula (8), we have

$$\widehat{\mathrm{E}}(\boldsymbol{Q}; \phi) = \frac{\sum_{j=1}^{M} \alpha_j(\phi)}{\sum_{j=1}^{M} \mathrm{I}(p_j < \alpha_j(\phi))}. \tag{12}$$

3 Algorithm for the quality control procedure

Algorithm 1: 'Outlier' Detection

Input: Measurements from evaluators, with study participants' characteristics data;
Output: Potential 'Outlier' evaluators ;
First stage: Run the no-intercept regression:
$\mathrm{E}(Y_i|\boldsymbol{X}_i, \mathrm{T}_i^{(1)}, \ldots, \mathrm{T}_i^{(M)}) = \sum_{j=1}^{M} \beta_j \mathrm{T}_i^{(j)} + \boldsymbol{\gamma}^T \boldsymbol{X}_i$;
Start of the second stage: Let Φ be a series of power values over the range of $(0, 1)$, e.g., $\Phi =$ `seq(from = 0.1, to = 0.95, by = 0.01)`
for *each* ϕ *in* Φ **do**
    for j *in* $1 : M$ **do**
        Derive the significance level $\alpha_j(\phi)$ corresponding to the test for $\widehat{\beta}_j$, based on either the truncated or untruncated test
    end
    Calculate the estimated FDR: $\widehat{\mathrm{E}}(\boldsymbol{Q}; \phi) = \frac{\sum_{j=1}^{M} \alpha_j(\phi)}{\sum_{j=1}^{M} \mathrm{I}(p_j < \alpha_j(\phi))}$
end
Create the FDR vs. Power plot by plotting ϕ versus the estimated FDR, and determine a reasonable power $\widetilde{\phi}$ based on the plot.
for j *in* $1 : M$ **do**
    if *p-value* $p_j < \alpha_j(\tilde{\phi})$ **then**
        Declare the j-th evaluator as an 'outlier'
    end
end
if *FDR-based adjustment* = *YES* **then**
    Let $k = \sum_{j=1}^{M} \mathrm{I}(p_j < \alpha_j(\widetilde{\phi}))$ and $\widehat{FDR} = \frac{\sum_{j=1}^{M} \alpha_j(\widetilde{\phi})}{\sum_{j=1}^{M} \mathrm{I}(p_j < \alpha_j(\widetilde{\phi}))}$;
    if $k * \widehat{FDR} > 1$ **then**
        Sort the declared 'outlier' evaluators using p-values in ascending order
        if *p-values of 'outliers' are of order* $k - \lceil k * \widehat{FDR} \rceil$ *to* k **then**
            Reclassify them as 'normal' evaluators
        end
    end
end
End of the second stage

4 Additional simulation results for repeated measurements

Data generation of repeated measurements

To illustrate the scenario when an evaluator measures multiple correlated outcomes within one study participant, we generate data obtained from two ears as follows:

$$\begin{aligned} \mu_{i,k} = \mathrm{E}(Y_{i,k}|\boldsymbol{X}_i) &= \gamma_1 \mathrm{Age}_i + \gamma_2 \mathrm{Age}_i^2 + \gamma_3 \mathrm{I}(\text{very good}_i) \\ &+ \gamma_4 \mathrm{I}(\text{a little hearing trouble}_i) + \beta_1 \mathrm{Audio}_i^{(1)} \\ &+ \beta_2 \mathrm{Audio}_i^{(2)} + \ldots + \beta_M \mathrm{Audio}_i^{(M)}, \\ (Y_{i,1}, Y_{i,2}) &\sim \mathrm{Normal}\left((\mu_{i,1}, \mu_{i,2}), \begin{bmatrix} \sigma^2 & \rho\sigma^2 \\ \rho\sigma^2 & \sigma^2 \end{bmatrix} \right), \end{aligned}$$

where the correlation coefficient ρ is 0.3, 0.5 and 0.8, reflecting mild, moderate and strong correlations. The parameter settings and variable generation mechanism remain the same as in the single measurement simulation.

Simulation results for repeated measurements

Supplementary Figure 1 shows the FDR vs. Power decision plots for the scenario when the correlation between two ears are 0.5. Compared with the single measurement simulation, the decision plots retain similar patterns, except that the estimated FDR tends to slightly overestimate the true value, yielding a more conservative outlier detection procedure.

Supplementary Figure 2, 5 and 6 show the true positive proportions for the 'outlier' Audiologists 1 to 8, and false positive proportions for the true 'normal' Audiologists 9 to 16, with $\sigma = 8, 10$ and 12. The simulation results are similar to the single measurement scenarios; our outlier detection procedure typically has lower false positive proportions for the true 'normal' audiologists and higher true positive proportions for the true 'outlier' audiologists compared with the approach that fix the significance level at $\alpha = 0.05$.

5 Supplementary Simulation Figures

5.1 One ear simulation

5.2 Two ear simulation with $\rho = 0.5$

5.3 Two ear simulation with $\rho = 0.3$

5.4 Two ear simulation with $\rho = 0.8$

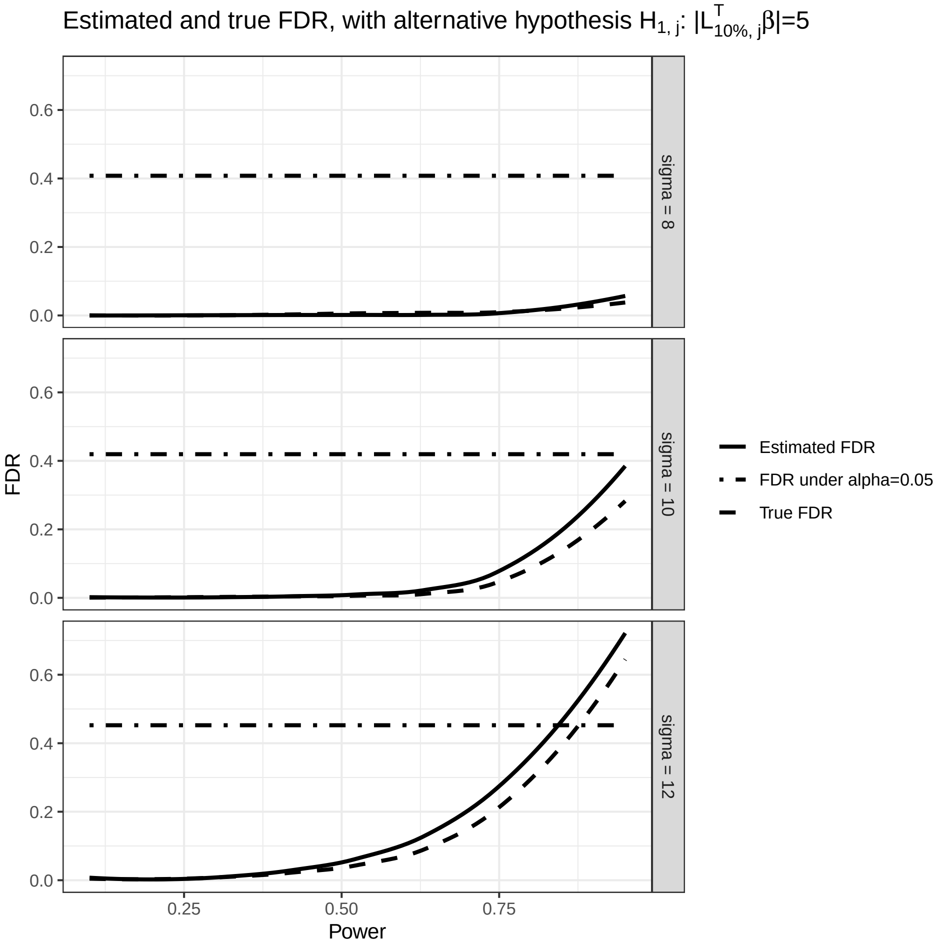

Figure 1: FDR vs. Power decision plot for multiple correlated measurements simulation with $\rho = 0.5$. The alternative hypothesis is $H_{1,j} : \left| \boldsymbol{L}_{10\%,j}^T \boldsymbol{\beta} \right| = 5$. The solid curve is the estimated FDR based on Equation (5) in the main document averaged over 300 simulation replicates, and the dashed curve is the empirical true FDR calculated by averaging the proportions of false discoveries $\frac{\boldsymbol{V}(\phi)}{\boldsymbol{R}(\phi)}$ over 300 simulation replicates. The black horizontal dot-dash line represents the empirical true FDR calculated by averaging the proportions of false discoveries over 300 simulation replicates when using $\alpha = 0.05$ as the significance level.

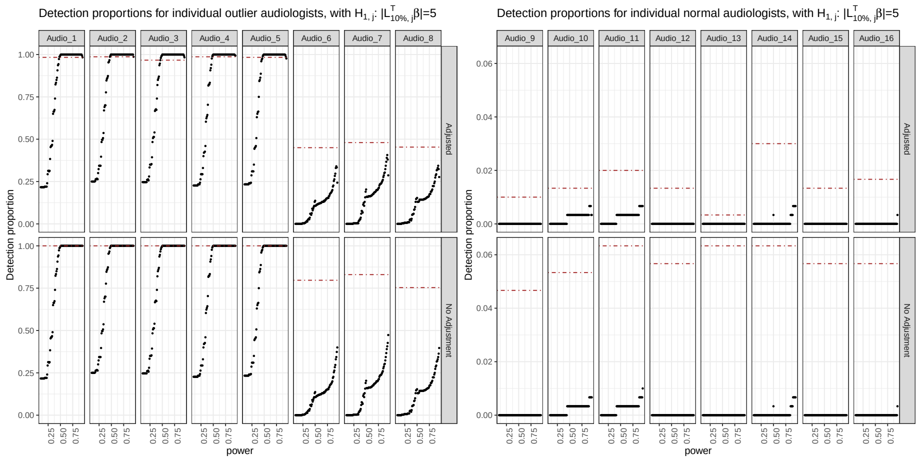

Figure 2: This figure shows the true positive proportions for the true 'outlier' audiologists and false positive proportions for the true 'normal' audiologists for the multiple correlated measurements simulation with the correlation coefficient $\rho = 0.5$ and $\sigma = 8$. The left figure shows the true positive proportions for Audiologists 1 - 8. The subfigure on the top is the result by performing the FDR-based adjustment, while the subfigure on the bottom is the result without FDR-based adjustment. The right figure shows the false positive proportions for Audiologists 9 - 16. The horizontal dot-dash line represents the corresponding true or false positive proportion for each audiologist if we use $\alpha = 0.05$ as the significance level for rejecting the null hypotheses.

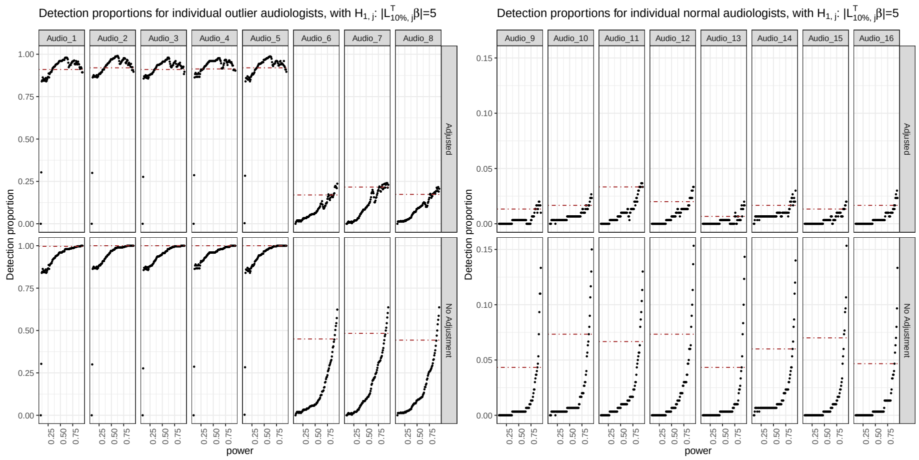

Figure 3: This figure shows the true positive proportions for the true 'outlier' audiologists and false positive proportions for the true 'normal' audiologists for single measurement simulation with $\sigma = 10$. The left figure shows the true positive proportions for Audiologists 1 - 8. The subfigure on the top is the result by performing the FDR-based adjustment, while the subfigure on the bottom is the result without FDR-based adjustment. The right figure shows the false positive proportions for Audiologists 9 - 16. The horizontal dot-dash line represents the corresponding true or false positive proportion for each audiologist if we use $\alpha = 0.05$ as the significance level for rejecting the null hypotheses.

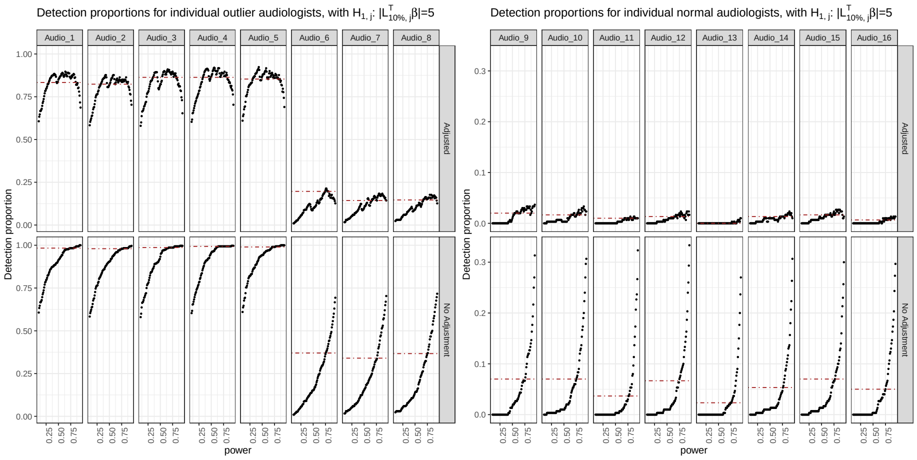

Figure 4: This figure shows the true positive proportions for the true 'outlier' audiologists and false positive proportions for the true 'normal' audiologists for single measurement simulation with $\sigma = 12$. The left figure shows the true positive proportions for Audiologists 1 - 8. The subfigure on the top is the result by performing the FDR-based adjustment, while the subfigure on the bottom is the result without FDR-based adjustment. The right figure shows the false positive proportions for Audiologists 9 - 16. The horizontal dot-dash line represents the corresponding true or false positive proportion for each audiologist if we use $\alpha = 0.05$ as the significance level for rejecting the null hypotheses.

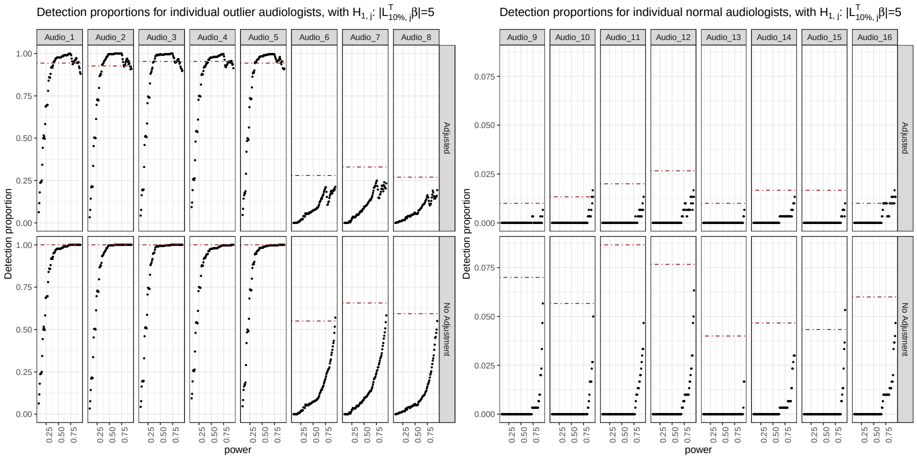

Figure 5: This figure shows the true positive proportions for the true ‘outlier’ audiologists and false positive proportions for the true ‘normal’ audiologists for the multiple correlated measurements simulation with the correlation coefficient $\rho = 0.5$ and $\sigma = 10$. The left figure shows the true positive proportions for Audiologists 1 - 8. The subfigure on the top is the result by performing the FDR-based adjustment, while the subfigure on the bottom is the result without FDR-based adjustment. The right figure shows the false positive proportions for Audiologists 9 - 16. The horizontal dot-dash line represents the corresponding true or false positive proportion for each audiologist if we use $\alpha = 0.05$ as the significance level for rejecting the null hypotheses.

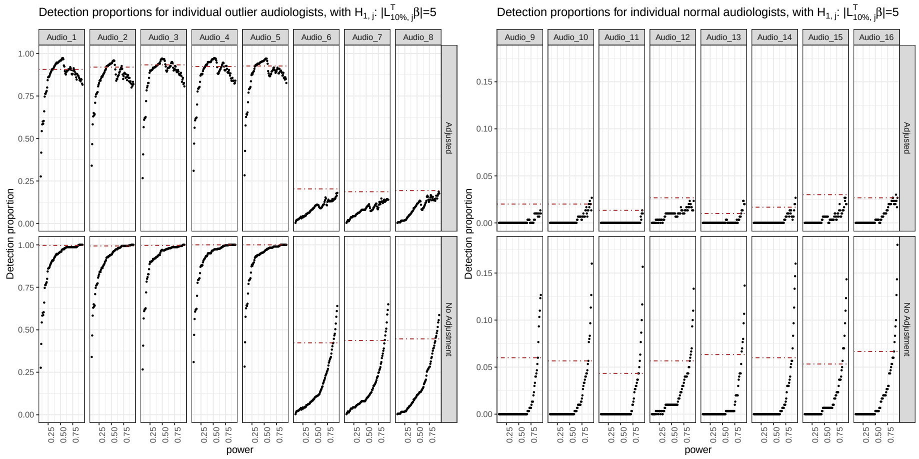

Figure 6: This figure shows the true positive proportions for the true 'outlier' audiologists and false positive proportions for the true 'normal' audiologists for the multiple correlated measurements simulation with the correlation coefficient $\rho = 0.5$ and $\sigma = 12$. The left figure shows the true positive proportions for Audiologists 1 - 8. The subfigure on the top is the result by performing the FDR-based adjustment, while the subfigure on the bottom is the result without FDR-based adjustment. The right figure shows the false positive proportions for Audiologists 9 - 16. The horizontal dot-dash line represents the corresponding true or false positive proportion for each audiologist if we use $\alpha = 0.05$ as the significance level for rejecting the null hypotheses.

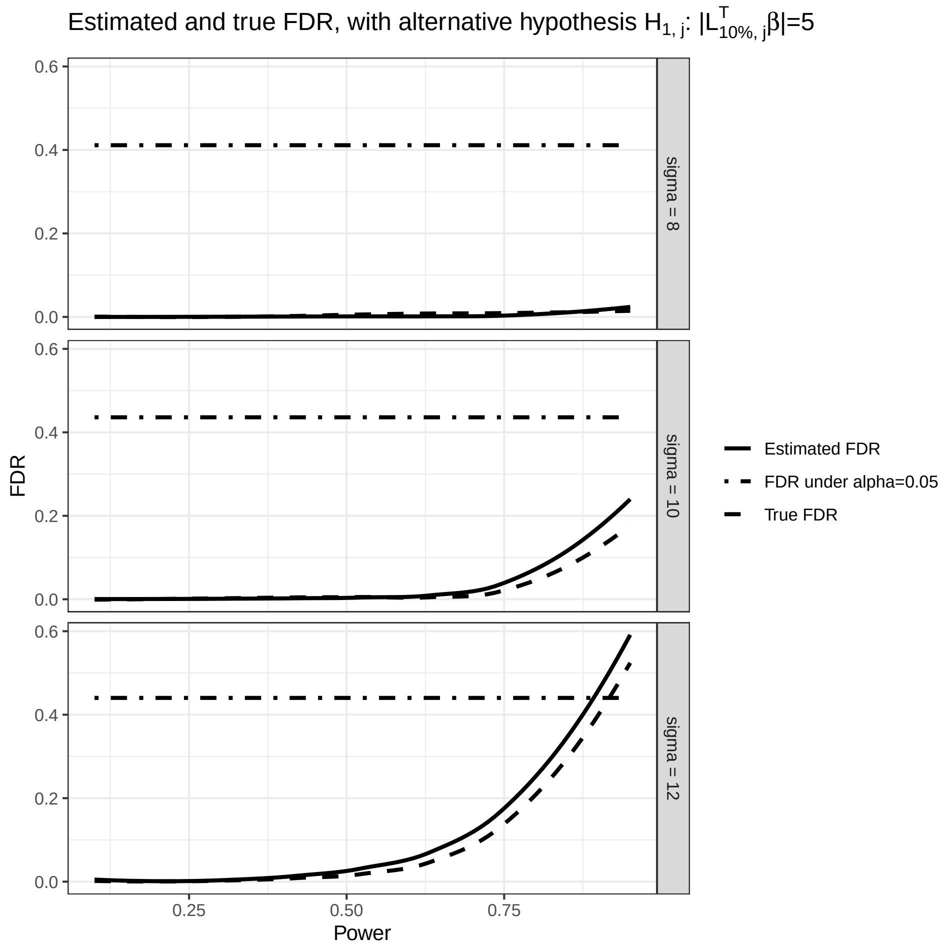

Figure 7: FDR vs. Power decision plot for multiple correlated measurement simulation with $\rho = 0.3$. The alternative hypothesis is $H_{1,j} : |\boldsymbol{L}_{10\%,j}^{T}\boldsymbol{\beta}| = 5$. The solid curve is the estimated FDR based on Equation(5) averaged over 300 simulation replicates, and the dashed curve is the empirical true FDR calculated by averaging the proportions of false discoveries $\frac{\boldsymbol{V}(\phi)}{\boldsymbol{R}(\phi)}$ over 300 simulation replicates. The black horizontal dot-dash line represents the empirical true FDR calculated by averaging the proportions of false discoveries over 300 simulation replicates when using $\alpha = 0.05$ as the significance level.

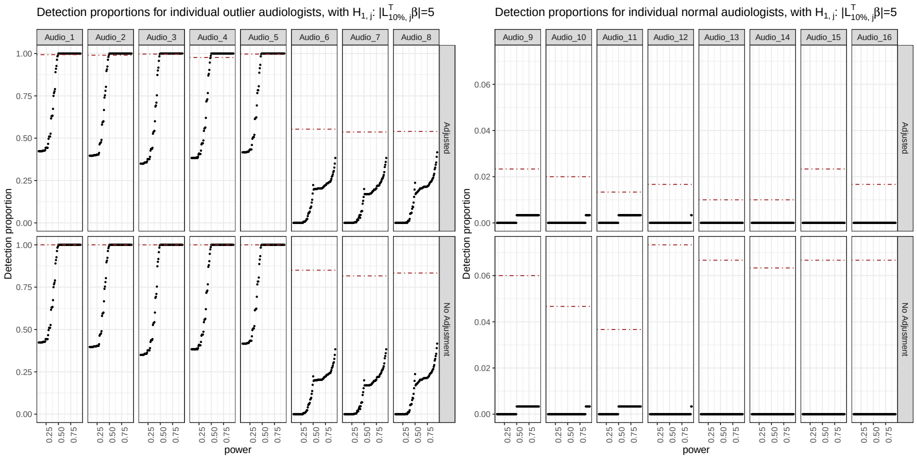

Figure 8: This figure shows the true positive proportions for the true 'outlier' audiologists and false positive proportions for the true 'normal' audiologists for the multiple correlated measurements simulation with the correlation coefficient $\rho = 0.3$ and $\sigma = 8$. The left figure shows the true positive proportions for Audiologists 1 - 8. The subfigure on the top is the result by performing the FDR-based adjustment, while the subfigure on the bottom is the result without FDR-based adjustment. The right figure shows the false positive proportions for Audiologists 9 - 16. The horizontal dot-dash line represents the corresponding true or false positive proportion for each audiologist if we use $\alpha = 0.05$ as the significance level for rejecting the null hypotheses.

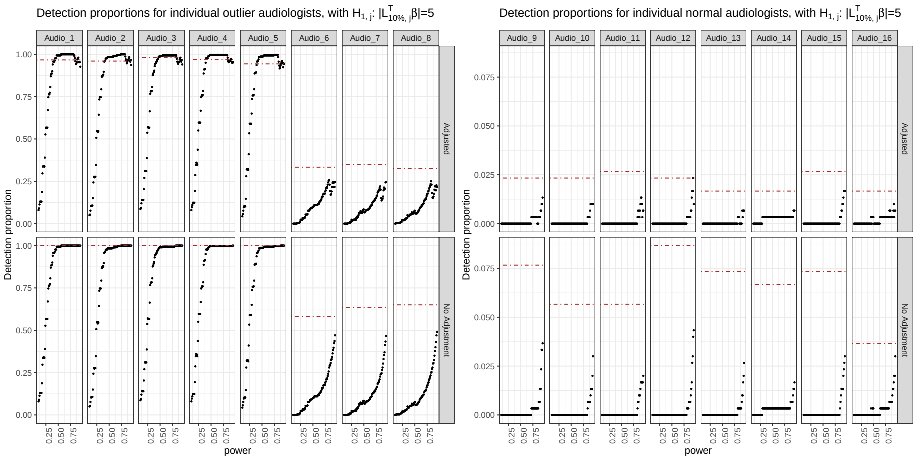

Figure 9: This figure shows the true positive proportions for the true 'outlier' audiologists and false positive proportions for the true 'normal' audiologists for the multiple correlated measurements simulation with the correlation coefficient $\rho = 0.3$ and $\sigma = 10$. The left figure shows the true positive proportions for Audiologists 1 - 8. The subfigure on the top is the result by performing the FDR-based adjustment, while the subfigure on the bottom is the result without FDR-based adjustment. The right figure shows the false positive proportions for Audiologists 9 - 16. The horizontal dot-dash line represents the corresponding true or false positive proportion for each audiologist if we use $\alpha = 0.05$ as the significance level for rejecting the null hypotheses.

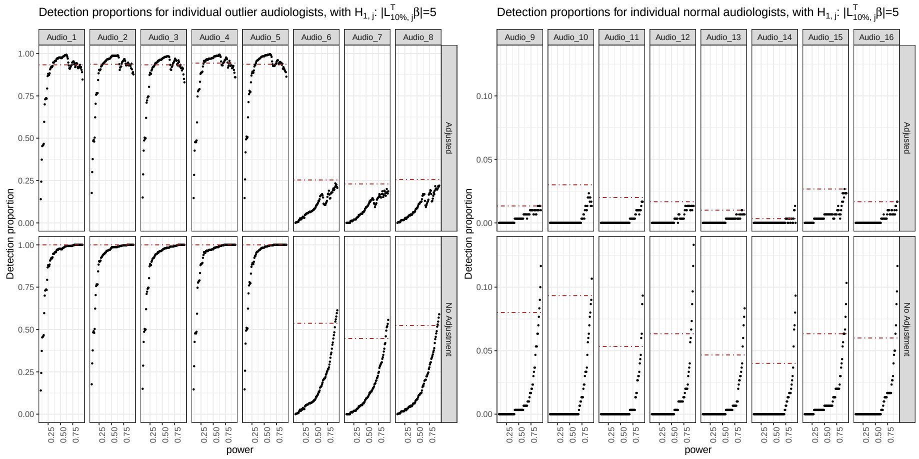

Figure 10: This figure shows the true positive proportions for the true 'outlier' audiologists and false positive proportions for the true 'normal' audiologists for the multiple correlated measurements simulation with the correlation coefficient $\rho = 0.3$ and $\sigma = 12$. The left figure shows the true positive proportions for Audiologists 1 - 8. The subfigure on the top is the result by performing the FDR-based adjustment, while the subfigure on the bottom is the result without FDR-based adjustment. The right figure shows the false positive proportions for Audiologists 9 - 16. The horizontal dot-dash line represents the corresponding true or false positive proportion for each audiologist if we use $\alpha = 0.05$ as the significance level for rejecting the null hypotheses.

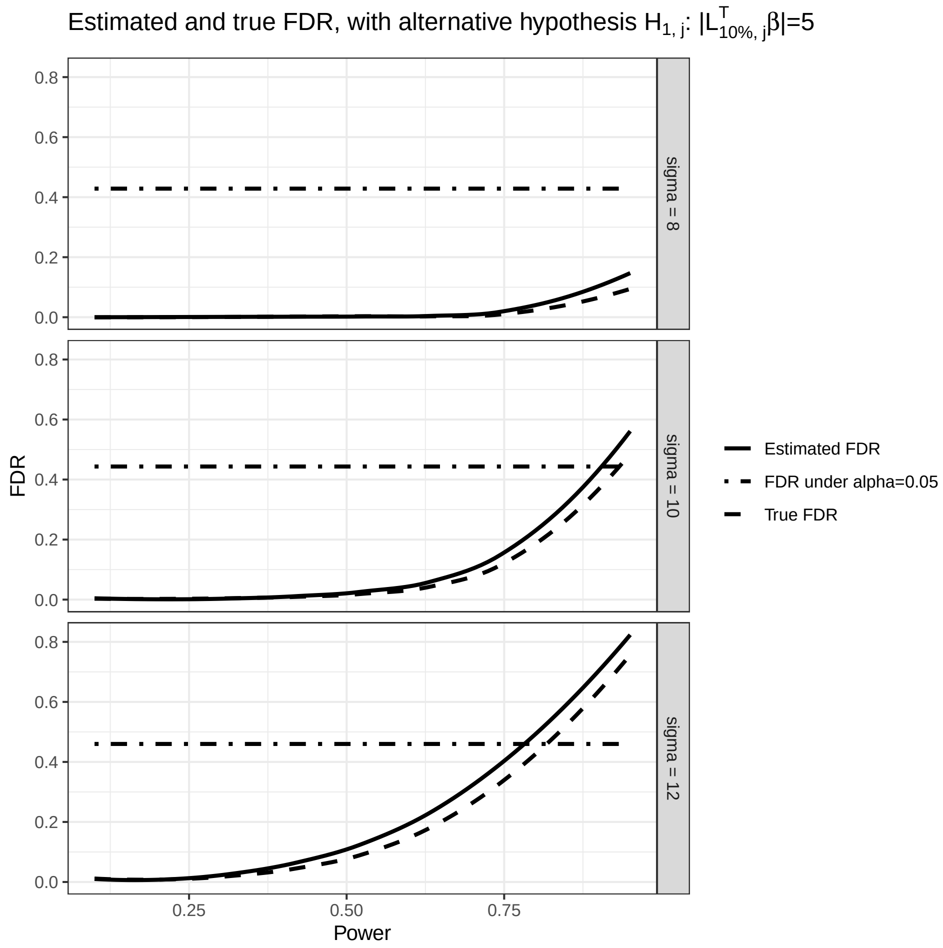

Figure 11: FDR vs. Power decision plot for multiple correlated measurement simulation with $\rho = 0.8$. The alternative hypothesis is $H_{1,j} : |\boldsymbol{L}^T_{10\%,j}\boldsymbol{\beta} = 5|$. The solid curve is the estimated FDR based on Equation (5) averaged over 300 simulation replicates, and the dashed curve is the empirical true FDR calculated by averaging the proportions of false discoveries $\frac{\boldsymbol{V}(\phi)}{\boldsymbol{R}(\phi)}$ over 300 simulation replicates. The black horizontal dot-dash line represents the empirical true FDR calculated by averaging the proportions of false discoveries over 300 simulation replicates when using $\alpha = 0.05$ as the significance level.

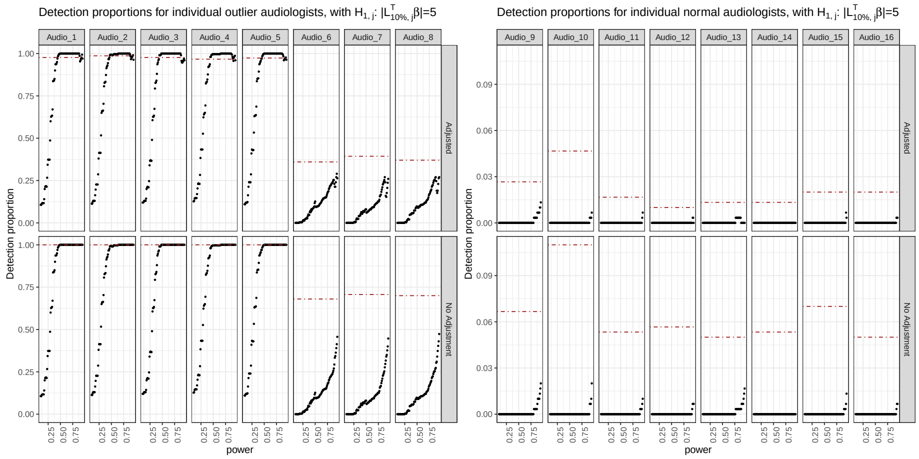

Figure 12: This figure shows the true positive proportions for the true ‘outlier’ audiologists and false positive proportions for the true ‘normal’ audiologists for the multiple correlated measurements simulation with the correlation coefficient $\rho = 0.8$ and $\sigma = 8$. The left figure shows the true positive proportions for Audiologists 1 - 8. The subfigure on the top is the result by performing the FDR-based adjustment, while the subfigure on the bottom is the result without FDR-based adjustment. The right figure shows the false positive proportions for Audiologists 9 - 16. The horizontal dot-dash line represents the corresponding true or false positive proportion for each audiologist if we use $\alpha = 0.05$ as the significance level for rejecting the null hypotheses.

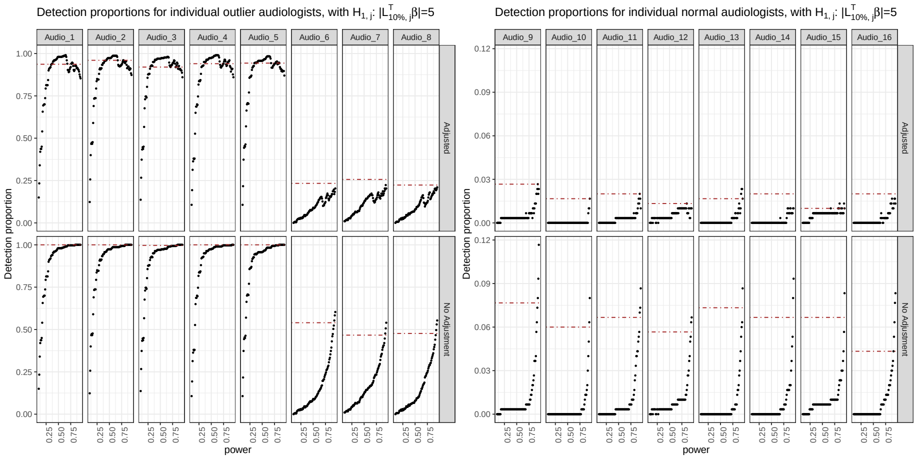

Figure 13: This figure shows the true positive proportions for the true 'outlier' audiologists and false positive proportions for the true 'normal' audiologists for the multiple correlated measurements simulation with the correlation coefficient $\rho = 0.8$ and $\sigma = 10$. The left figure shows the true positive proportions for Audiologists 1 - 8. The subfigure on the top is the result by performing the FDR-based adjustment, while the subfigure on the bottom is the result without FDR-based adjustment. The right figure shows the false positive proportions for Audiologists 9 - 16. The horizontal dot-dash line represents the corresponding true or false positive proportion for each audiologist if we use $\alpha = 0.05$ as the significance level for rejecting the null hypotheses.

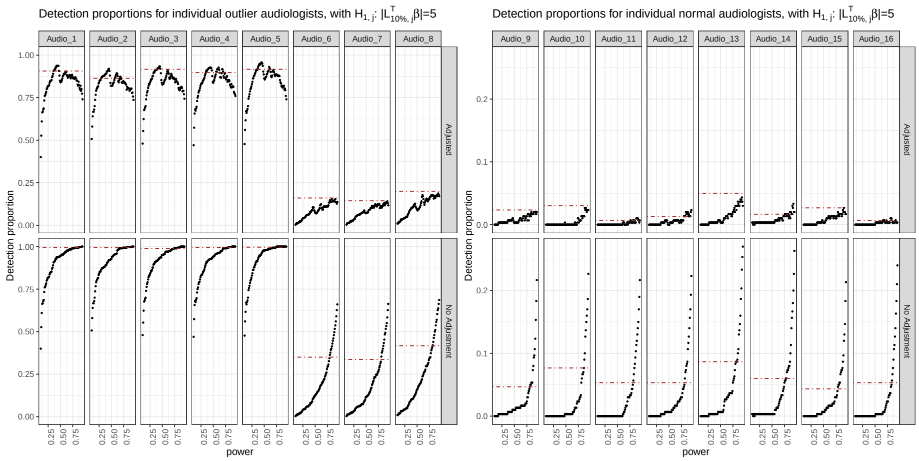

Figure 14: This figure shows the true positive proportions for the true 'outlier' audiologists and false positive proportions for the true 'normal' audiologists for the multiple correlated measurements simulation with the correlation coefficient $\rho = 0.8$ and $\sigma = 12$. The left figure shows the true positive proportions for Audiologists 1 - 8. The subfigure on the top is the result by performing the FDR-based adjustment, while the subfigure on the bottom is the result without FDR-based adjustment. The right figure shows the false positive proportions for Audiologists 9 - 16. The horizontal dot-dash line represents the corresponding true or false positive proportion for each audiologist if we use $\alpha = 0.05$ as the significance level for rejecting the null hypotheses.
